# A Biobank for Long-term and Sustainable Research in the Field of Congenital Heart Disease in Germany

**DOI:** 10.1016/j.gpb.2016.03.003

**Published:** 2016-04-27

**Authors:** Thomas Pickardt, Eva Niggemeyer, Ulrike M.M. Bauer, Hashim Abdul-Khaliq, Competence Network for Congenital Heart Defects Investigators

**Affiliations:** 1 National Register for Congenital Heart Defects, 13353 Berlin, Germany; 2 Saarland University Medical Center, Department of Paediatric Cardiology, 66421 Homburg, Germany; 3 Competence Network for Congenital Heart Defects, 13353 Berlin, Germany

**Keywords:** Congenital heart defects, Multi-center research, Biorepository, Genetic research, DNA

## Abstract

Congenital heart disease (CHD) is the most frequent birth defect (0.8%–1% of all live births). Due to the advance in prenatal and postnatal early diagnosis and treatment, more than 90% of these patients survive into adulthood today. However, several mid- and long-term morbidities are dominating the follow-up of these patients. Due to the rarity and heterogeneity of the phenotypes of CHD, multicenter registry-based studies are required. The CHD-Biobank was established in 2009 with the aim to collect **DNA** from patients and their parents (trios) or from affected families, as well as cardiovascular tissues from patients undergoing corrective heart surgery for cardiovascular malformations. Clinical/phenotype data are matched to the International Paediatric and Congenital Cardiac Code (IPCCC) and the International Statistical Classification of Diseases and Related Health Problems 10th Revision (ICD-10). The **DNA** collection currently comprises samples from approximately 4200 participants with a wide range of CHD phenotypes. The collection covers about 430 trios and 120 families with more than one affected member. The cardiac tissue collection comprises 1143 tissue samples from 556 patients after open heart surgery. The CHD-Biobank provides a comprehensive basis for research in the field of CHD with high standards of data privacy, IT management, and sample logistics.

## Introduction

Congenital heart disease (CHD) is the most common congenital organ malformation in the neonate. The prevalence of CHD is nearly stable and ranges from 8–10/1000 [1], [2]. Due to the rapid improvement in prenatal and postnatal early diagnosis, as well as the advance in interventional, surgical, and postoperative treatment, the survival of offsprings with CHD has increased dramatically in the last two decades [3], [4], [5], [6]. Nevertheless, in moderate and severe congenital heart defects, anatomical correction could not be achieved. Intracardiac and extracardiac anatomical and functional residual defects which lead to mid- and long-term morbidities are still challenging and limit the long-term survival and quality of life of these patients [7].

The etiology of CHD, which is complex and associated with both genetic and environmental causes, is still largely unknown [8], [9]. With the growing number of adult CHD patients, information on recurrence risks and understanding the etiology will become increasingly important. Despite the introduction of novel genetic techniques such as next-generation sequencing (NGS), the molecular and genetic mechanisms underlying the development of CHD have not been clearly elucidated yet and the identification of particular defects remains challenging [10], [11].

Moreover, in contrast to the intensive research in the field of ischemic heart failure and the development of therapeutic and preventive approaches in adult patients, the therapeutic and preventive strategies for heart failure in patients with CHD are not adequately established. The molecular mechanisms of myocardial failure in neonatal myocardium after corrective surgery using the cardiopulmonary bypass (CPB), as well as in patients with residual pressure and volume overload, have not been clearly identified yet [12].

The rarity of such congenital diseases and their heterogeneous morphological phenotypic and morphological manifestation make it difficult to study molecular and genetic origin as well as prevention and treatment [13]. Therefore, CHD and its surgical correction are still associated with high mortality in the neonatal life, in contrast to older age groups [14], [15].

Although the neonatal myocardium may still have protective remodeling capacities such as stem cell and myocyte proliferation [16], [17], the vulnerability of the neonatal myocardium to ischemia and other risk factors during the surgical manipulation and extracorporeal perfusion by CPB may represent significant risk factors [18], [19], leading to myocardial dysfunction after heart surgery. On the other hand, residual adaptive mechanisms of the fetal heart such as cell proliferation and the presence of stem cells have been considered as possible adaptive capacities against stress and ischemic injury [16], [17]. Data on the remodeling processes in these patients during the transition from fetal to neonatal life are scarce and should be evaluated within the biobank on the basis of a standardized collection of myocardial tissues.

Thus, the establishment of a nationwide research platform to overcome such challenges in the most common congenital organ malformations in the future is urgently needed. Moreover, extensive research cooperation should not be limited to nationwide research groups, but should also be extended to other countries and performed on an international level, as demonstrated by our network during the past five years (see below, section “Cooperative projects and partner institutions”).

## Network and biobank infrastructure

**Figure 1:**
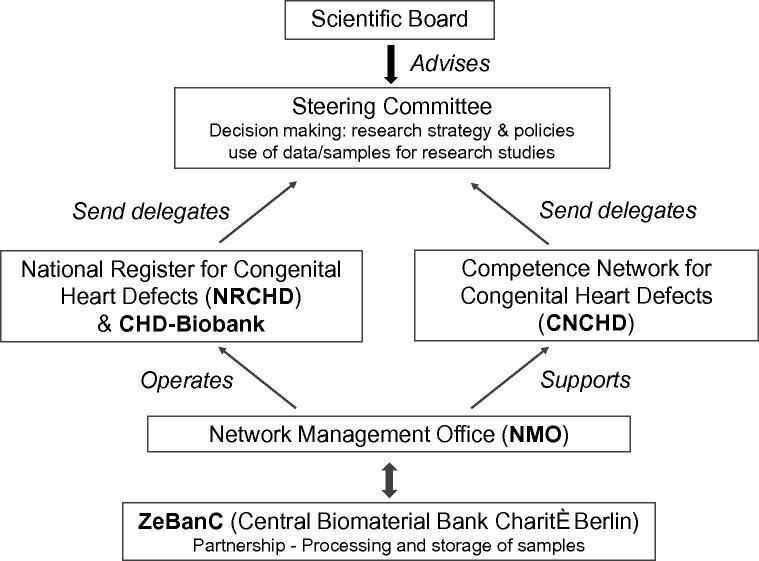
Structure of CNCHD and NRCHD

The CHD-Biobank is an integral part of the National Register for Congenital Heart Defects (www.kompetenznetz-ahf.de/en/research/register-biobank, hereafter NRCHD), which serves as a core facility of the German Competence Network for Congenital Heart Defects (www.kompetenznetz-ahf.de/en/home, hereafter CNCHD). Both entities are non-profit organizations and operate independently of each other, thus guaranteeing the separation of sustainable and long-term patient data administration and storage on the one hand and research activities on the other hand (Figure 1). The management and coordination for both entities are performed by a central office, the Network Management Office (NMO). The NMO is responsible for patient registration, administration, and consent management. It is also responsible for sample collection, validation of clinical/phenotype data, and research/study management, as well as network communication and operation of database systems. The whole network is headed by a Steering Committee that includes board members from both NRCHD and CNCHD. An essential element of the CHD-Biobank infrastructure is the cooperation with the Central Biomaterial Bank Charité Berlin (ZeBanC, biobank.charite.de) that carries out central processing of blood and saliva samples and storage of DNA.

While numerous medical centers and institutions are involved in various studies of the CNCHD, the CHD-Biobank project is maintained by eight institutions: the departments of pediatric cardiology and/or heart surgery at the University Hospitals of Kiel, Erlangen, Freiburg, and Homburg, the University Heart Center of Leipzig, the Heart and Diabetes Center NRW (Bad Oeynhausen), the German Heart Centre Munich, and the German Heart Institute Berlin. Further inclusion of more hospitals is scheduled. Participation is bound to the implementation of all required workflows, and the participating institutions confirm the acceptance of CHD-Biobank policies and standard operation procedures (SOPs), based on a collaboration agreement.

## Study population

The CHD-Biobank collects biomaterials from patients with any diagnosed congenital heart defect. For reference analyses, samples of patients’ parents (trios) and relatives (from families with an accumulation of CHD) are also included systematically.

## Legal framework—ethics and data privacy

Based on an appropriate patient informed consent, approval by eight ethics committees in Germany and a data privacy concept (registered with the Berlin Official for Data Protection and Freedom of Information/No. 531.390), NRCHD is legally authorized to (i) receive and store medical data and samples from all age groups for an indefinite time, (ii) continuously request latest medical reports from the attending physicians, (iii) re-contact registered participants, (iv) hold the right to use the data and samples collected, and (v) make register data and samples available to interested scientists and research institutions for future studies and within the scope of international cooperation.

### Protection of personal data

Primary patient codes (pseudonyms) are kept in a separate database (PID-generator, see below) to prevent unauthorized access to re-identifying information. All samples are tagged with a second-level code that is designated only for internal use, *i.e.*, the processing and storage at the collaborating laboratory facility, where staff members are not able to obtain information on the identity of sample donors or their health conditions. When samples or aliquots of samples are released to research facilities, a third-level code is generated, impeding the re-identification of patients.

### The use of data

The use of data by research institutions is subject to the CNCHD policies and a material transfer agreement (MTA) that commits data recipients to handle data according to legal regulations and applicable laws, *e.g.*, preventing third parties from having access to or using samples and data.

### Patients’ right of self-determination

The storage of samples is generally intended for an indefinite time and yet-to-define research purpose. In order to give patients the opportunity to obtain a continuous update regarding the CHD-Biobank’s research activities (and to withdraw their consent, if required), the NRCHD has implemented a web-based patient information platform (www.herzregister.de). Sample donors can object to the CHD-Biobank’s storing and using their data/samples at any time. Donors can opt between the anonymization and deletion/destruction of data/samples.

## Database systems and ID management

In consistence with the NRCHD’s overall concept, the CHD-Biobank applies a central patient and sample ID management. Patient codes are generated by a so-called PID-generator [20], a software program that creates 8-digit/alphanumeric pseudonyms on the basis of the patients’ personally-identifiable information. The latter is divided into hard and soft criteria and phonetic algorithms, thus ensuring the unambiguity of a patient’s data upon pseudonymization. The PID-generator allows a hospital-independent assignment of patients and facilitates multicenter and longitudinal investigations.

For clinical data and sample acquisition, the NRCHD uses a 3-tier server architecture consisting of Oracle 10g, which is a customized version of ixserv4 (ixmid Technologie GmbH, www.ixmid.com), as the application layer, and a Tomcat web server for the presentation layer. The database for biomaterials includes a web frontend with a defined form structure that enables the acquisition of sample-related data (reception, processing, quality, dissemination, tracking, *etc*.) and facilitates the collection and management of samples from several locations. Sample ID management is performed using uniform barcode-labeling systems including barcoded sample containers with unique alphanumeric codes.

## Clinical data

Clinical data for each participant are obtained via medical reports (doctor’s letters) that are provided by the treating physicians or by the patients themselves. A standardized dataset is recorded from each patient upon registration. The recorded data include (1) personal data: surname, birth name, first name, date of birth, sex, address and contact details, familiar status and relations (acquisition of families), place of birth including federal state, marital status, and nationality, and (2) medical/phenotype data: maternal age at birth, gestational age, multiple pregnancy, birth weight, prenatal diagnosis of CHD, all congenital and acquired cardiovascular diagnoses, procedures, and additional diseases/disorders. If necessary for accuracy and data validation, further medical findings, *e.g.*, additional data of magnetic resonance imaging (MRI)/echocardiographic analysis, are requested from the treating physicians. In addition, medical reports are obtained regularly to continuously update/complete the medical database.

Medical data are coded according to (i) the International Statistical Classification of Diseases and Related Health Problems 10th Revision (ICD-10) and (ii) the International Paediatric and Congenital Cardiac Code (IPCCC) of the International Society for Nomenclature of Pediatric and Congenital Heart Disease comprising 206 different main diagnoses for CHD (Table 1) in order to cover the high variability of CHD phenotypes and conditions. For a rough classification we assign affected sample donors to 29 diagnosis groups (see below, section “Current state of sample collection”).

## Procedures for the collection, processing and storage of samples

The CHD-Biobank collects EDTA-blood (Sarstedt S-Monovette K3E 2.7/4.9/9.0 ml, depending on donor age) and saliva samples (DNAGenotek/Oragene OG-500 and OG-575) for the extraction of DNA, as well as cardiac tissue samples (snap-freezing in liquid nitrogen). The latter are obtained within the scope of open heart surgery during which fractions of tissue accumulate, which would be disposed of otherwise.

### Blood/saliva collection and DNA extraction

Two means of sample acquisition are in place. **Process A** is used for the acquisition of blood and saliva samples from patients that are already registered, and their relatives. Patients are contacted by the NMO and asked for participation in the CHD-Biobank project. Adult patients or sample donors, after returning written consent, receive a blood or saliva sample kit. For blood taking, sample donors address a physician of their choice. Blood or saliva samples are sent to ZeBanC. Process A is most suitable for recruiting families and twins via direct contact to participants of the NRCHD. On the other hand, **Process B** is used for the acquisition of blood samples from patients not yet taking part in the NRCHD. Collaborating hospitals are regularly provided by the NMO with blood kits that include coded documents (informed consent and sample data sheets) and barcoded sample containers that are linked to the respective documents (recorded in the CHD-Biobank database). Within the scope of scheduled examinations, patients are informed and blood withdrawal takes place if they agree. The responsible physician forwards the signed consent form together with a medical report to the NMO, and the blood sample to ZeBanC.

A small amount of blood sample is retained for control purposes and stored at −80 °C. Saliva and the remaining blood samples are further processed at ZeBanC. For the automated extraction of DNA from blood and saliva, ZeBanC uses the Freedom EVO HSM 2.0 Workstation and the ReliaPrep Large Volume HTgDNA Isolation System (Promega/Tecan). The processing, aliquoting, and storage of samples are documented via the online-accessible CHD-Biobank database. The documented data include information with respect to the date of blood taking, dispatch and arrival, blood volume/quality, extraction-related information/specifications, DNA purity, gel-quality check, concentration, volume and aliquotting. DNA is stored at −20 °C.

### Tissue samples from open heart surgery

**Figure 2:**
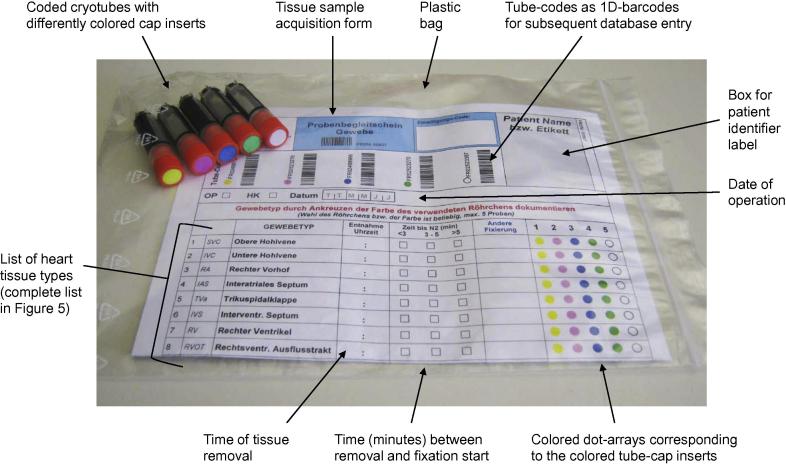
Tissue-sample collection kit The complete list of heart tissue types can be found in Figure 5.

The NMO provides specific tissue sample kits (Figure 2) for all participating departments. A sample kit comprises five cryotubes (FluidX, 1.8 ml), each of which is provided with a unique identifier (2D + 1D barcodes), and a standardized sample acquisition form, tagged with the identifiers of the above tubes, printed as 1D-barcodes. Tubes and acquisition form are therefore already linked prior to their usage. Moreover, the acquisition form contains a list of heart tissue types that have been pre-defined by the Network surgeons.

When tissue kits are allocated to a certain patient, the surgical team can collect tissue samples without the need of further marking/labeling tubes. The type of tissue that is placed in one of the five tubes is readily designated: tube caps contain inserts in different colors which are reproduced as dots alongside the list of tissue types; then the corresponding dot just has to be ticked on the acquisition form.

Samples are snap-frozen in liquid nitrogen and then stored in the gas phase of liquid nitrogen. The samples are stored in local facilities at the participating medical centers. All sample data (vial code, type of tissue, acquisition date, position in the nitrogen-storage container) are entered electronically via web interface into the CHD-Biobank database.

## Sample quality management

All workflows have been defined in SOPs and are revised and adjusted continuously according to technological progress. SOPs conform to or are based on the International Society for Biological and Environmental Repositories (ISBER) recommendations (http://www.isber.org). The following measures have been introduced in order to maintain high quality standards regarding the logistics and infrastructure of the CHD-Biobank.

### Uniform protocols and workflows

The NMO team provides an efficient data and sample management, the implementation of harmonized protocols and workflows, standardized logistics, efficient central administration, and a continuous risk assessment.

### Guideline-based patient contact

Recruitment of patients and families is accompanied by substantial guideline-based telephone interviews performed by NMO staff members that are specifically instructed and allowed to initiate and hold contact to register participants.

### On-site visits

Participating hospitals are controlled through on-site visits by NMO staff members at least once a year, including the instruction of hospital staff members involved in data and sample collection, as well as documentation.

### Permanent availability and support

The NMO team permanently keeps in touch with the responsible hospital staff members and provides them with support.

### Web-based data entry

Information relevant to sample quality, acquisition, and processing procedures is recorded in the online-accessible CHD-Biobank database. The NMO team monitors data entry in real time.

### Use of 1D/2D barcodes

For nitrogen storage of samples, FluidX Cryovials with 2D-barcode jackets are used. The identification and recording of samples occur via barcode reading (linear- and 2D-barcode readers) only.

### Secure nitrogen storage and handling

For tissue storage, the CHD-Biobank is equipped with Cryotherm Biosafe 120 MD containers providing an online-accessible, permanent all-over control and complete documentation of temperature and nitrogen filling status. For incorporating and disseminating samples, a working procedure preventing thawing and refreezing has been implemented.

## Network policies and use of the sample collection

The NRCHD is a sustainable legal entity that is eligible to receive, store, and use data and samples from patients with CHD for research purposes. The sample donors transfer the right to use their biomaterials for scientific purposes to the NRCHD, which reserves the right to use as long as sample donors do not withdraw their consent.

Use and access regulations and policies (see www.kompetenznetz-ahf.de/en/research/initiating-new-projects) comprise (i) general rules for cooperative studies, (ii) defined forms/templates for research proposals, (iii) a defined description of the decision process, and (iv) publication guidelines.

The CHD-Biobank offers researchers both the option of using existing samples and associated demographic and medical data, as well as the option of utilizing the CHD-Biobank infrastructure for a prospective recruitment of sample donors for specific studies. This service is open to all researchers or scientists with relevant expertise in the field of genetics and CHD and is not limited to members of the CNCHD.

In order to participate, researchers have to submit a research proposal to the NMO. Research proposals are evaluated by the Scientific Board*,* which consists of experts in the fields of (pediatric) cardiology, epidemiology, human genetics, basic research, biometry/statistics, and ethics/health care law. Moreover, the Board comprises a representative of the German umbrella organization for children with CHD (BVHK e. V.). The final decision is made by the Steering Committee of the CNCHD and the representatives of the institutions where the samples have been collected, based on criteria such as a project’s feasibility, the precisely-defined objective, acceptance within the CHD research community, and stringent scheduling. Appropriate expertise in the field of interest is therefore a prerequisite.

Upon approval, collaborating research groups and institutions must agree to make, after completion and publication of their study, exploitable data such as sequence data available for being used by research groups in future studies.

## Sustainability

Since January 2015, the NRCHD is supported by the newly established German Center for Cardiovascular Research (DZHK). Within this close cooperation, a comprehensive sustainable research network could be established, which covers congenital and acquired cardiovascular diseases in children and adults.

## Current state of sample collection

Table 2 gives an outline of the current DNA collection of the CHD-Biobank (as of March 2016): the collection comprises DNA samples from 4602 donors, including 2928 affected donors, covering a wide range of CHD phenotypes. 1674 donors are non-affected relatives, since the CHD-Biobank focuses on the inclusion of whole families when more than one affected member is present, and the enrollment of trios (affected children and their healthy parents). In addition to family and trio samples, the inclusion of twins is crucial for research on etiology and genetic factors of CHDs as well. The CHD-Biobank recruits families and twins mainly by direct contact to registered participants via Process A (as described in section “Blood/saliva collection and DNA extraction”).

**Figure 3:**
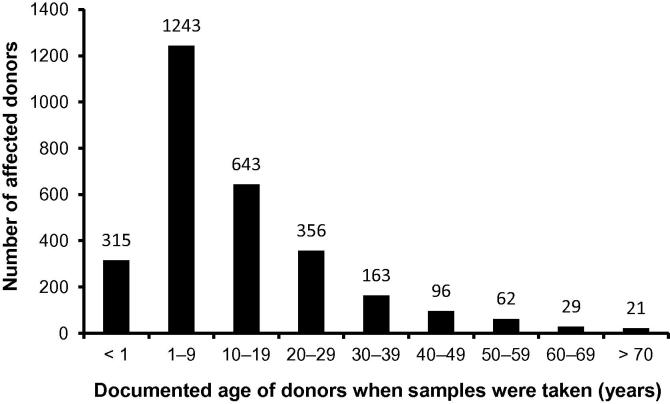
Age distribution of the affected donors The age here refers to the documented age when samples were taken.

The breakdown of sample donors by age groups (Figure 3, documented age when samples were taken) reveals the predominance of underage patients (0–17 years), which represent more than 70% of the affected donors.

Table 3 shows the distribution of the 29 diagnosis groups that we use for rough classification of the affected donors. The distribution does not reflect the average distribution of CHDs, since the CHD-Biobank focuses mainly on medium or severe, complex heart defects.

**Figure 4:**
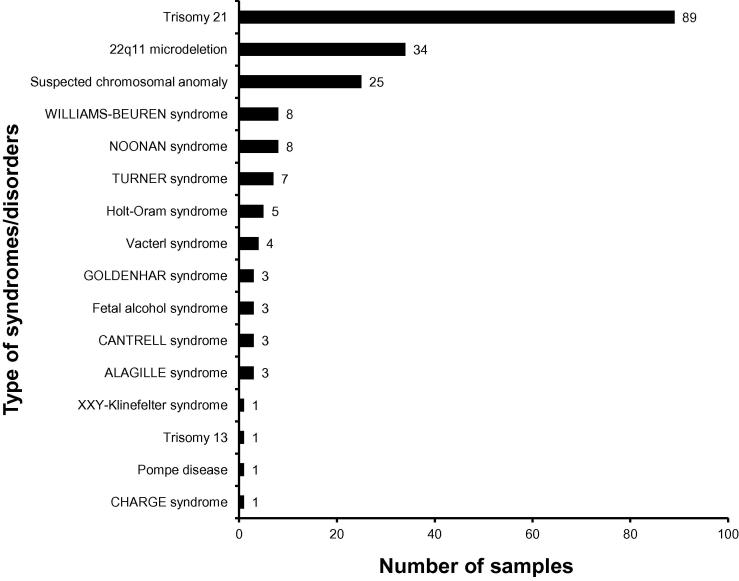
Distribution of the affected donors born with additional syndromes/disorders

While the majority of children born with CHDs do not have other birth defects (isolated CHD), 20%–30% of cases occur in association with other, extracardiac anomalies or as part of a syndrome (syndromic CHD). Figure 4 shows the distribution of syndromes/disorders among the affected donors in the CHD-Biobank.

For DNA extraction, 9 ml of blood are withdrawn from adults. In acordance with the German ethical guidelines regarding underage participants, amounts of maximally 9 ml (age 3–17), 5 ml (age 1–3), and 2.5 ml (below age 1) of blood are taken. Moreover, the ethics commission specifies that blood to be used for scientific purposes may only be taken from underage patients within the scope of a medical examination. We therefore additionally implemented saliva sample kits (DNAGenotek, Oragene, standard amount of 2 ml saliva) for DNA acquisition from affected, underage donors beyond medical examinations and for non-affected siblings.

The automated extraction method for EDTA-blood on average yields 15.2 μg DNA from 1 ml EDTA-blood and 16.8 μg DNA from 1 ml of saliva. In addition to the routine control via gel electrophoresis and absorbance ratios 260/280 and 260/230 nm, the quality of DNA from 1147 sample donors has been checked within the scope of the cooperative project with the Wellcome Trust Sanger Institute (WTSI, Cambridge/UK). In more than 98% of samples, whole exome- or panel-sequencing was successful.

**Figure 5:**
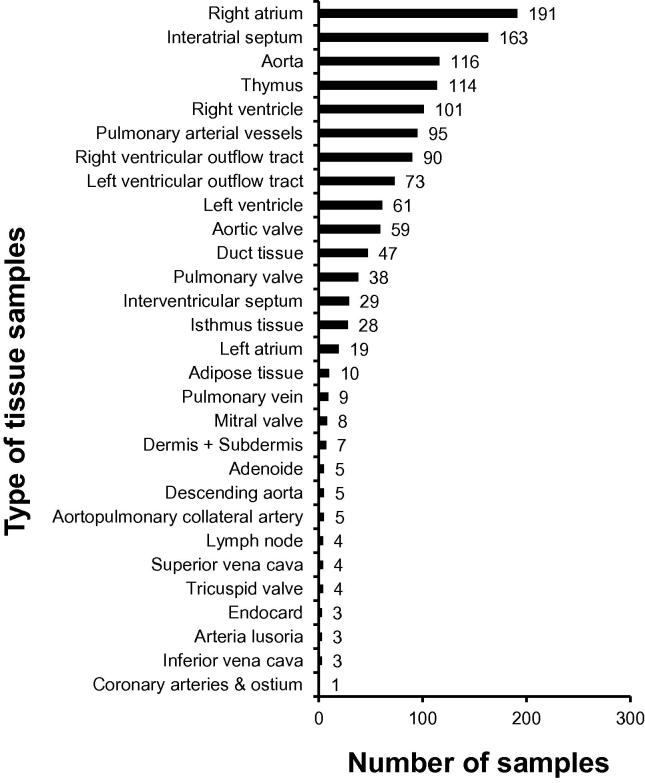
Type and number of tissue samples from heart surgery

Figure 5 shows the tissue samples that have been collected so far at the heart surgery departments of the university hospitals of Kiel, Erlangen, and Saarland/Homburg, as well as the German Heart Institute in Berlin. In total, 1295 tissue samples have been collected from 659 patients, which are listed in Figure 5 according to the tissue type. An RNA quality control analysis of 20 tissue samples within the scope of our cooperative project with the Department of Pediatric Cardiology and Critical Care at Hannover Medical School revealed RNA integrity number (RIN) values of 8.2–9.4, indicating a high quality of the RNA generated.

## Cooperative projects and partner institutions

At the time when the CHD-Biobank was initiated (2008/09), research on CHD genetics was only slightly established in Germany, partially due to the limited availability of samples. We therefore started cooperative projects on an international level from the beginning. A number of projects have since then been completed and further projects are in progress or in preparation. Projects include candidate gene approaches, chip-based copy number variation (CNV) analysis, and various NGS approaches. Up to now, a total of 1588 DNA samples of the affected and non-affected donors, as well as 36 tissue samples from heart surgery, have been provided for various research studies (Table 4).

## Conclusions

Research activities including molecular mechanisms of the development of cardiovascular malformations and several associated long-term morbidities are increasingly becoming a substantial subject of genetic studies, largely due to sequencing capacities being available and a more routine use of bioinformatic analysis techniques.

The identification of the causes and underlying molecular genetic processes, and an improved understanding of the pathologic mechanisms of long-term morbidities, are the prerequisites of developing new prevention strategies and treatment methods for patients with CHD. In particular, for patients with moderate and severe CHD in their early life, morbidities after successful operative treatment are now challenging their long-term survival and quality of life. Unfortunately, there are only scarce data on molecular mechanisms underlying myocardial systolic and diastolic dysfunction and possible therapeutic targets in the left and right ventricle. Similarly, data from patients having a morphologically single ventricle with heterogeneous anatomy are rare as well. The genetic mechanisms underlying several extracardiac morbidities, including hypoplasia and inadequate pulmonary growth in patients with restricted pulmonary perfusion, rapid growth and dilatation of the ascended aorta in patients with univentricular heart or tetralogy of Fallot and pulmonary atresia, are currently unknown. Thus, prospective clinical and genetic trials in larger cohorts are needed. These can only be performed on the basis of a nationwide registry and biobank.

The CHD-Biobank includes comprehensive representative genetic materials of patients and their parents as well as further relatives, covering severe and mild CHD. The Network Management Team is currently increasing the efforts to include more families with accumulation of congenital cardiomyopathies.

The rising number of requests for collaboration from numerous national and international research institutions can be regarded as an indication for the high quality of samples and phenotype data deposited in our biobank.

The described biobank may represent a future platform for prospectively recruiting cohorts of patients with complex CHD, including the comprehensive phenotypic and genetic characterization in early childhood, as well as repeated clinical and cardiac follow-up and assessments at standardized time intervals. Such an approach may be the basis to develop individualized medical treatment and care for patients with these rare and complex CHDs.

## Authors’ contributions

TP, EN, UMMB, and HAK have made equal contributions to the biobank concept and design, and preparation of the manuscript. HAK is the Speaker of the Network and UMMB is the Managing Director of the Network. All authors have read and approved the final manuscript.

## Competing interests

The authors declare that there are no competing interests.

## Figures and Tables

**Table 1:** Comparison between ICD-10 and IPCCC for the classification of congenital heart defects

**Diagnosis/treatment**	**Code range for ICD-10**	**Code range for IPCC**
Main diagnosis	70	206
Congenital cardiac secondary diagnosis	69	204
Acquired cardiac secondary diagnosis	108	218
Hereditary, fetal and neonatal diagnosis	42	55
Extracardiac diagnosis	All available codes	N/A
Cardiac operations	N/A	299
Cardiac interventions	N/A	66

*Note:* ICD-10, International Statistical Classification of Diseases and Related Health Problems 10th Revision; IPCCC, International Paediatric and Congenital Cardiac Code. ICD-10 codes overlap between different categories.

**Table 2:** Outline of the current CHD-Biobank DNA collection

**Participant classification**		**Number**
Donors	Affected donors	2928
	Non-affected donors	1674

Families with	2 affected members	102
	3 affected members	24
	4 affected members	3

Trios (patient plus healthy parents)		453
Enrolled twin pairs	Monozygotic	15
	Dizygotic	19
	Status still unknown	13

**Table 3:** Distribution of the affected donors by diagnosis groups

**Diagnosis group**		**Number of the affected donors**
1	Aortic coarctation	243
2	Aortic valve abnormalities	201
3	Atrioventricular septal defect	177
4	Common arterial trunk	32
5	Complete transposition of great arteries	105
6	Congenitally corrected transposition of great arteries	61
7	Discordant VA connections	55
8	Double inlet left ventricle	73
9	Double outlet right ventricle	89
10	Ebstein’s malformation of tricuspid valve	49
11	Hypoplastic left heart syndrome	189
12	Interatrial communication	345
13	Interrupted aortic arch	11
14	MARFAN Syndrome	11
15	Mitral atresia	9
16	Mitral valve abnormalities	21
17	Partially anomalous pulmonary venous connection(s)	45
18	Patent arterial duct	115
19	Primary Cardiomyopathy	80
20	Pulmonary atresia + intact ventricular septum	40
21	Pulmonary atresia + ventricular septal defect	124
22	Pulmonary valve abnormalities	79
23	Shone Complex	9
24	Subaortic stenosis	12
25	Tetralogy of Fallot	279
26	Total anomalous pulmonary venous connection	13
27	Tricuspid atresia	80
28	Ventricular septal defect	227
29	Other	154

**Table 4:** Cooperative projects and partner institutions

**Partner institutions**	**Sample type**	**CHD type /study**	**No. of samples**	**Status**	**Refs.**
Academic Medical Centre Amsterdam, the Netherlands; Institute of Human Genetics, Newcastle University, UK; Heart Repair-Consortium	DNA	Complex CHD	78	Completed	[21], [22]
Department of Pediatric Cardiology, Friedrich-Alexander-Universität Erlangen-Nürnberg	DNA	CM	62	Completed	[23]
Department of Congenital Heart Disease and Pediatric Cardiology, University Hospital of Schleswig-Holstein, Campus Kiel, and further international partner institutions	DNA	CM	16	Completed	[24]
Department of Cardiovascular Genetics, Experimental and Clinical Research Center, Charité – Universitätsmedizin Berlin and Max Delbrück Center (MDC) for Molecular Medicine, Berlin, Germany	DNA	TOF families	35	Completed	[25]
Wellcome Trust Sanger Institute, Cambridge/UK, and further international partner institutions	DNA	AVSD	18	Completed	[26]
Department of Pediatric Cardiology, Friedrich-Alexander-Universität Erlangen-Nürnberg	DNA	CoA	83	Completed	[27]
Institute of Biochemistry and Molecular Genetics, Ulm University	DNA	Heterotaxy	74	Completed	Manuscript submitted
Department of Cardiology and Pneumology, Göttingen University	DNA	HLHS	72	In progress	
Department of Pediatric Cardiology and Critical Care, Hannover Medical School	Heart tissue	TOF	36	In progress	
Wellcome Trust Sanger Institute, Cambridge/UK and further international partner institutions	DNA	LVOTO	1147	Completed	Manuscript submitted

*Note:* CHD, congenital heart disease; CM, primary cardiomyopathy; TOF, tetralogy of Fallot; AVSD, atrioventricular septal defect; CoA, aortic coarctation; HLHS, hypoplastic left heart syndrome; LVOTO, left ventricular outflow tract obstruction.
